# Phytochemical Characterization, Antioxidant and In Vitro Cytotoxic Activity Evaluation of *Juniperus oxycedrus* Subsp. *oxycedrus* Needles and Berries

**DOI:** 10.3390/molecules24030502

**Published:** 2019-01-30

**Authors:** Reda Ben Mrid, Najat Bouchmaa, Youssef Bouargalne, Btissam Ramdan, Khalid Karrouchi, Imad Kabach, Miloud El Karbane, Abderrazak Idir, Abdelmajid Zyad, Mohamed Nhiri

**Affiliations:** 1 Laboratory of Biochemistry and Molecular Genetics, Faculty of Sciences and Technologies of Tangier, BP 416, 90000 Tangier, Morocco; rbenmrid@gmail.com (R.B.M.); youssef.bouargalne@gmail.com (Y.B.); warda_dan_123@hotmail.com (B.R.); imad.kabach@gmail.com (I.K.); 2 Team of Experimental Oncology and Natural Substances, Cellular and Molecular Immuno-pharmacology, Faculty of Science and Technologies, Sultan Moulay Slimane University, 23000 Beni-Mellal, Morocco; najat.bouchmaa@gmail.com (N.B.); idir.abdou.92@gmail.com (A.I.); ab.zyad2@gmail.com (A.Z.); 3 Physicochemical service, Drugs Quality Control Laboratory, Division of Drugs and Pharmacy, Ministry of Health, 10100 Rabat, Morocco; 56.khalid@gmail.com; 4 Laboratory of Analytical Chemistry and Bromatology, Faculty of Medicine and Pharmacy, Mohamed V University, 10100 Rabat, Morocco; elkarbane76@yahoo.fr

**Keywords:** *Juniperus oxycedrus* subsp. *Oxycedrus*, human breast cancer, cytotoxicity, oxidative stress, antioxidant activities

## Abstract

In order to evaluate the antioxidant properties of aqueous and methanol extracts of needles and berries of *Juniperus oxycedrus* subsp. *oxycedrus* (*Joo*) species, various antioxidant capacity assessment tests (free radical scavenging assays (DPPH• and ABTS•+ tests), ferrous ions (Fe^2+^) chelating activity and reducing power assay (FRAP) were conducted. In all of the tests, the extracts exhibited strong antioxidant activity. Furthermore, in-vitro cytotoxic activity assays of the methanolic extracts showed potent cytotoxic effects against two breast cancer cell lines (MDA-MB-468 and MCF-7), with no cytotoxicity towards normal cells (PBMCs). Reactive oxygen species generation was presumed to be a potential reason for the observed cytotoxic effects. According to all the above, and considering its appropriate composition of mineral elements and phenolic compounds, *Joo* could offer a beneficial and natural source of bioactive compounds that can be either used on the preventive side as it could potentially be used in the clinic without toxicity.

## 1. Introduction

Among the different human cancers, breast cancer is the leading cause of mortality in both developing and developed countries [1]. The incidence of breast cancer is constantly increasing [1], leading to increased economic costs and an onus shared among women and their families.

It is well known that the increase of reactive oxygen species (ROS) production or the decrease of the antioxidant defense system efficiency is responsible for cells’ oxidative stress [2]. Numerous studies have suggested the implication of ROS in the underlying molecular mechanisms involved in all the steps of carcinogenesis (initiation, promotion and progression) [2]. Furthermore, Khan et al. have suggested a link between antioxidant and anticancer activity [3]. Thereby, antioxidants are proposed as potential candidates for both the prevention and treatment of cancer [4]. For this reason, several synthetic antioxidants and anticancer drugs have been proposed by pharmaceutical companies. However, using such synthetic compounds has several side effects [4]. As an alternative, scientists have resorted to natural molecules present in fruits, vegetables and herbs, usually safe for human consumption, which could be considered as strong antioxidant and anti-proliferative molecules.

The *Juniperus* L. genus is a member of the Cupressaceae family. The genus comprises about 70 species which are distributed throughout the Northern Hemisphere [5]. Despite their use in the treatment of several diseases, such as tuberculosis, pneumonia, bronchitis, diarrhea, stomach aches, and hyperglycemia, there are few studies concerning chemical composition and biological activity of the *Juniperus* L. genus [5]. From the entire *Juniperus* genus, *J. oxycedrus* is one of the most renowned species used in folk medicine [6].

Previous studies have reported cytotoxic effects of some *Juniperus* species on some human cancer cell lines such as lung cancer A549 cells [7]. However, to the best of our knowledge, this is the first report on the cytotoxic effect of *Juniperus oxycedrus* L. subsp. *oxycedrus* against two human breast cancer cell lines, MDA-MB-468 and MCF-7. To do this, the present study aimed, on the one hand, to investigate the chemical composition and the antioxidant capacity of methanolic and aqueous extracts of *Juniperus oxycedrus* subsp. *oxycedrus* (*Joo*) needles and berries. On the other hand, the methanolic extracts of both organs were evaluated for their cytotoxic effects against triple-negative breast cancer (TNBC) subtype line (MDA-MB-468) and the luminal subtype line (MCF-7).

## 2. Results and Discussion

### 2.1. Identification and Quantification of Phenolic Compounds

Table 1 shows the composition and concentration of the major phenolic compounds in the needles and berries of *Joo*.

Eleven phenolic compounds were grouped as phenolic acids, flavonoids and terpenes and were identified or not according to their retention times and UV spectra which were compared to the corresponding commercial standards (Figure S1). The levels of these compounds were higher in the needles compared to berries. The hydroxybenzoic acids were the most abundant compounds in the needles. However, in the berries, low amounts of phenolic acids were detected. Moreover, this chemical identification showed different profiles between the methanolic and the aqueous extracts. The most abundant compound in needles was salicylic acid (3398.1 mg/100 g and 2942.7 mg/100 g for aqueous and methanolic extracts respectively), followed by rutin (1080.5 mg/100 g and 160.6 mg/100 g for aqueous and methanolic extracts respectively). In the berries, the most abundant compound was hesperidin (147 mg/100 g) followed by salicylic acid (128 mg/100 g) for the aqueous extract and rutin for the methanolic extract (60 mg/100 g). No salicylic acid was detected in the methanolic extract of the *Joo* berries. Furthermore, the GA, SyA and limenone were not detected in all the extracts.

Only a few studies have been conducted to analyze the chemical composition of the *Juniperus* genus and even fewer have focused on *Joo*. In a previous study, rutin was the main component of the methanolic extract of *Joo* needles (11.02 mg/100 g) and only 1.0 mg/100 g was observed for *Joo* berries [7]. In *J. communis* var. saxatilis, rutin was reported to be the most abundant compound (1220 mg/100 g), however, the content of common phenolic acids was low, with total hydroxybenzoic acids amounting up to 34 mg/100 g and total hydroxycinnamic acids up to 26 mg/100 g [8]. Considering the fact that there are only few data concerning the chemical composition of *Joo*, the present results are of great importance and the identified molecules can be added to the known molecules identified in this species.

### 2.2. Mineral Composition of Joo Needles and Berries

The mineral elements are divided in plants into macroelements and microelements. These elements are implicated in important biological functions in the cell. The elemental composition of *Joo* needles and berries is given in Table 2. The macroelements (Ca, K, Mg, Na and P) and microelements (Co, Fe, Mn, Zn, Cr, Cu and Se) were determined in both *Joo* plant organs. Our results clearly indicated that Ca is the most abundant macroelement in both needles and berries. The concentration of this element ranged between 20.19 g/kg in the needles and 4.61 g/kg in the berries. Potassium was the second most abundant element with a concentration of 7.95 g/kg in the needles and 2.78 g/kg in the berries. For the other macroelements, concentrations ranged from 4.54 to 3.41 g/kg for Mg, 2.31 to 2.42 g/kg for Na and 1.68 to 1.61 for P in needles and berries respectively. The levels obtained for Ca, Mg and Na was higher compared to those obtained for *J. phoenicea* berries (0.95 g/kg, 0.65 g/kg and 0.64 g/kg respectively) [9]. However, the content of K obtained for *J. phoenicea* berries was higher (3.74 g/kg) compared to our results.

The concentrations of the microelements ranged from 285.7 mg/kg mg to 59.66 mg/kg for Fe, 97.47 mg/kg to 59.01 mg/kg for Zn, 79.91 mg/kg to 29.78 mg/kg for Mn, 1.94 mg/kg to1.92 mg/kg for Cu, 0.993 to 1.28 mg/kg for Se and 0.354 to 0.366 for Co in needles and berries, respectively. Cd was not detected and Cr was detected only in leaves with a concentration of 0.602 mg/kg (Table 2). The results obtained by Ozkaya et al. for *J. oxycedrus* L. seeds are in agreement with our results concerning only the content of Mn (27.79 mg/kg). However these authors obtained higher levels of Cu (7.10 mg/kg), Cr (2.87 mg/kg) and Fe (187.95 mg/kg). Concerning Zn, the concentration obtained by these authors (7.70 mg/kg) was lower compared to our results [10].

Due to their high content of macoelements and the suitable amounts of trace elements, the needles and berries of *Joo* can be suggested as healthy nutrition. Moreover, the absence/very low concentrations of Cd, Cu, Cr and Se in the needles and berries of *Joo* is of great importance for their clinical use without toxicity.

### 2.3. Total Phenolic and Flavonoid Contents

Plants with high levels of secondary metabolites, such as phenolic and flavonoid compounds, are characterized by an important antioxidant activity. These secondary metabolites were reported to have therapeutic properties on several diseases like cancer [5]. Phytochemical analysis of *Joo* showed different levels of phenolic compounds between needles and berries and between aqueous and methanolic extracts (Table 3). In fact, the highest content of phenolic compounds was found in the methanolic extract of *Joo* needles (292.5 mg GAE/g dw), while the lowest level was obtained in the aqueous extracts of berries (28.1 mg GAE/g dw). Concerning flavonoids, their highest concentration was registered in the methanolic extract of the needles (54.6 mg QE/g dw) followed by the aqueous extract of the same organ (28.7 mg QE/g of dw). In berries, the total flavonoid content ranged from 3.2 mg QE/g dw for the aqueous extract to 8.3 mg QE/g dw for the methanolic extract.

Comparing our results with those obtained for other species of the *Juniperus* genus, we observed that *Joo* berries have higher levels of phenolic compounds compared to *J. phoenicea* berries which showed only 38.86 mg GAE/g dw and 49.43 mg GAE/g dw for aqueous and methanolic extracts respectively [11]. In the needles of *J. sibirica* Burgsdorf, the content of total phenolic compounds was 163.66 mg GAE/g dw for needles and 62.13 mg GAE/g dw for berries. The flavonoid content in the needles of *J. sibirica* Burgsdorf was also lower than the level we actually obtained [5]. The differences between these results may be attributed either to the species, the extraction method, or to the geographical environments and the development stage of the plant.

### 2.4. Antioxidant Activity

To validate the hypothesis of the antioxidant activity of *Joo* extracts, different in-vitro assays were conducted to measure the antioxidant capacity of both needles and berries: free radical scavenging assays (DPPH• and ABTS•+ tests), ferrous ions (Fe^2+^) chelating activity and reducing power assay.

Both methanolic and aqueous extracts of needles and berries displayed free radical scavenging activity (Table 3). Based on the IC_50_ values, the methanolic extract of the needles showed the highest ability to scavenge DPPH• free radicals followed by the methanolic extract of the berries (0.05 mg/mL and 0.09 mg/mL, respectively). In a study conducted by Taviano et al., the authors obtained almost the same results for the methanolic and aqueous extracts of *Joo* branches compared to our results obtained for needles (46 and 136 µg/mL, respectively) [12]. However, the IC_50_ value obtained for berries extract was much lower compared to the study conducted by Taviano et al. who obtained an IC_50_ of 1.5 mg/mL for the methanolic extract of *Joo* [13]. As already mentioned, the differences between results could be due to the differences in the extraction methods as it can be related to the geographical environments and the development stage of the plant.

The ability of *J. oxycedrus* extracts to scavenge free radicals was also examined by their capacity to quench ABTS•+. The results represented in the Table 3 showed that the methanolic extract of the needles exhibited the highest activity with an IC_50_ = 0.12 mg/mL followed by the methanolic extract of the berries (IC_50_ = 0.30 mg/mL). The Aqueous extracts have the lowest activity with an IC_50_ of 0.46 mg/mL and 1.64 mg/mL for the needles and berries, respectively (Table 3). These results confirm the high ability of needles to scavenge ABTS•+ compared to the berries. Furthermore, this strong antioxidant activity is in agreement with a previous work in which the ability to quench ABTS•+ of the methanolic extract of *Joo* needles was evaluated and showed an IC_50_ of 0.09 mg/mL [14].

The Fe^2+^ chelating activity is of great importance for the estimation of the antioxidant activity of the plant extracts. In fact, the transition metal ion Fe^2+^ is considered as the most potent lipid oxidation pro-oxidant because of its high reactivity [15]. Higher levels of ferrous ions contribute to the oxidative damage which can lead to various anomalies in the body. Therefore, the chelation of this metal ion could be an effective tool to prevent the oxidative damage. The present study showed that both needles and berries have chelating activity and the highest IC_50_ value was obtained for the aqueous extract of the berries (IC_50_ = 0.96 mg/mL). For the needles, the methanolic extract exhibited higher Fe^2+^ chelating activity compared to the aqueous extract (Table 3). In a previous study, Taviano et al. evaluated the Fe^2+^ chelating activity of the methanolic extract of *Joo* berries and obtained an IC_50_ of 2.61 mg/mL, which is higher compared to our results (1.89 mg/mL) [13].

The FRAP assay depicts electron donating capacity of bioactive molecules thereby allow the determination of their reducing power [16]. The results obtained for the FRAP test showed different reducing capacity towards FE^3+^-TPTZ complex when comparing the methanolic and aqueous extracts of the needles and berries of *Joo* (Table 3). Thus, the methanolic extracts showed always the highest reducing power. Moreover, the reducing power was extremely higher in the berries (941.81 mg AAE/g dw) compared to the needles (139.14 mg AAE/g dw). This result is not in line with the one obtained by Lesjak et al. who found that the reducing power of *J. sibirica* Burgsdorf. extracts was higher in the needles (114.81 mg AAE/g dw) compared to the berries (35.26 mg AAE/g dw) [5].

*Joo* needles and berries extracts exhibited strong antioxidant capacity which is in line with the results obtained previously for *Juniperus* extracts [5,11,13,14]. Furthermore, the observed antioxidant capacity of *Joo* extracts is related to their high content of phenolic and flavonoid metabolites. More specifically, the phenolic compounds determined by HPLC could explain the antioxidant capacity of *Joo* needles and berries since they are well-known for their antioxidant activities [17]. Therefore, the presence of such phenolic compounds in the daily diet could improve the cellular antioxidant defenses. In addition to this, these molecules may also have potent therapeutic activities that should be investigated.

On the other hand, we observed that the needles have greater amounts of phenolic and flavonoid compounds than the berries. This explains the higher antioxidant capacity of the needles compared to berries. However, the FRAP assay showed different results. In fact, the reducing power was significantly higher in the berries compared to the needles. Moreover, Table S1 showed negative correlation between the reducing power and the phenolic and flavonoids contents, suggesting that in *Joo* needles and berries other constituents contributed to this antioxidant activity [18]. Furthermore, the results presented here have shown a strong reverse correlation between the content of phenolic and flavonoid contents and the IC_50_ values of DPPH and ABTS (Table S1). It can therefore be concluded that *Joo* needles and berries can be considered as good food ingredients with high free radical-scavenger compounds and thus, high antioxidant activity.

### 2.5. Cytotoxicity of Methanolic Extracts of Joo Needles and Berries Against MDA-MB-468 and MCF7 Cell Lines

An increased number of studies concerning the effects of *Juniperus* species extracts have reported the importance of this species as source of natural molecules with potent anti-cancer activity [7,13]. In the current research, the cytotoxic activity of *Joo* needles and berries was evaluated toward two human breast carcinoma cell lines, MDA-MB-468 and MCF-7, using the MTT assay. As reported in Figure S2, the effect of both extracts was dose-dependent and was higher in cells treated with the methanolic extract of berries compared to needles. The IC_50_ was calculated for each extract and Table 4 showed that the lowest IC_50_ value was related to the MCF-7 treated cells (5.23 µg/mL and 10.75 µg/mL for berries and needles respectively). Concerning the MDA-MB-468 cell line, the IC_50_ was 6.43 µg/mL for the berries and 14.26 µg/mL for the needles.

Different therapeutic effects were reported for the methanolic extracts of *Joo* berries and needles, among of them, we can point out the antioxidant, antifungal, anti-inflammatory and acethylcholinesterase inhibitory activities [6,13]. Concerning their antiproliferative effects, the methanolic extracts of *Joo* and *J. oxycedrus* subsp. *macrocarpa* (*Jom*) ripe berries from Turkey were evaluated against the human hepatocellular liver carcinoma (HepG2) cell line [13]. However, the results obtained showed that both *Joo* and *Jom* extracts have no effect on HepG2 cell viability after treatment for 24 h [13]. In another study, De Marino et al. [19] tested the effect of an *n*-butanol extract of *Joo* berries on three human cancer cell lines, MCF-7, (breast), A375 (malignant melanoma) and H460 (lung). The results obtained showed that the extract decreased the cell viability only in MCF-7 cells.

The primary intention of cancer chemotherapy is to specifically target cancer cells without displaying toxicity toward normal cells. In the present study, the toxicity on non-cancerous cells putted into consideration. Hence, we also evaluated the cytotoxicity of the *Joo*’s needles and berries extracts on human normal peripheral blood mononuclear cells (PBMC) (Figure S3). Both needles and berries extracts showed low cytotoxicity on PBMC cells with an IC_50_ ≥ 49 µg/mL. According to the American National Cancer Institute (NCI), the criteria of a crude extracts of herbs to exhibit cytotoxic effect is an IC_50_ < 30 μg/mL. These findings suggest a high selective killing ability of these extracts for tumor cell lines (MDA-MB-468 and MCF7) without impacting normal cells. Recall that PBMCs are the first normal cell populations that come into contact with antitumor drugs used in conventional chemotherapy and that collapse from the first week of intravenous treatment of patients resulting in significant immune deficiency and increased side effects.

### 2.6. Clonogenic Assay of MDA-MB-468 and MCF-7 Treated with Methanolic Extracts of Joo Needles and Berries

**Figure 1:**
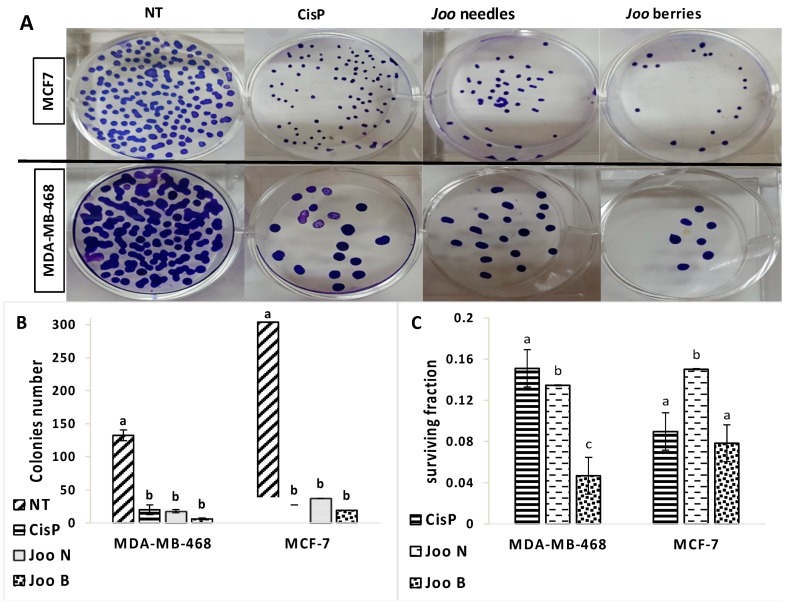
Clonogenic assay performed on MDA-MB-468 and MCF-7 cells treated with 1 µg/mL of *Joo* neeedles or berries extract or cisplatin (0.1 µg/mL) for 10 days. (**A**) Sample plate images from the clonogenic assay; (**B**) total number of colonies formed; (**C**) survival fraction. Each value represents the mean ± standard deviation of three independents measurements. Different letters in the same column indicate significant differences (*p* < 0.001) within conditions according to one-way ANOVA multiple comparison range Test.

Clonogenic cells compose a small portion of tumor cells within a tumor mass with the capacity for self-renaissance. Therefore, one of the frequent deficiencies in cancer therapies is the lack of eradication of all cells capable of re-growing the tumor. The clonogenic assay is a well-established in vitro cell survival test based on the capacity of a single cell to originate a colony. The clonogenic assay was performed to confirm the potential of *Joo* extracts to suppress the growth of MDA-MB-468 and MCF-7 cell lines. As shown in Figure 1, *Joo* extracts extensively reduced the clonogenic ability of both cell lines. Even so, this effect was more pronounced in MDA-MB-468 cells compared to the MCF-7 cells. Moreover, in both cell lines, the effect of the berries extract was more pronounced. The ability to reduce the formation of viable colonies in the MDA-MB-468 cells more effectively than the MCF-7 cell line indicates that the colony formation by these extracts is not mainly due to their extensive cell death.

### 2.7. Effect of Methanolic Extracts of Joo Needles and Berries on Antioxidant Enzyme Activities in MDA-MB-468 and MCF-7 Tumors Cell Lines

**Figure 2:**
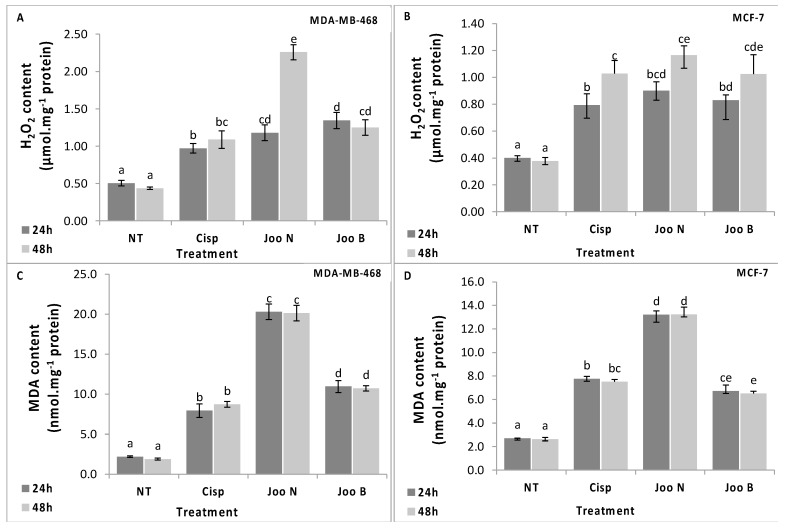
H_2_O_2_ content (**A** and **B**) and MDA content (**C** and **D**) in the MDA-MB-468 (**A** and **C**) and MCF-7 (**B** and **D**) cell lines after treatment with methanolic extracts of *Joo* needles (*Joo* N) and *Joo* berries (*Joo* B). Each value represents the mean of six replicates. Bars represent the standard error. Different letters indicate significant differences among treatments at *p* = 0.05.

Cancer cells as compared to normal cells are under greater intrinsic oxidative stress due to their alterations in metabolism. Furthermore, it was reported that cancer cells have an elevated level of ROS compared to normal cells. This elevation of ROS may be the result of an abnormal mitochondrial oxidative metabolism and can be responsible for the initiation and progression of different types of cancer such as breast, prostate, lung and colon cancers [3,20]. However, the elevation of ROS to a certain level may be lethal for tumor cells themselves. In fact, several studies outlined the implication of elevated levels of H_2_O_2_ in the induction of apoptosis [3] and their low concentration enhanced proliferation and migration of mammalian cells [20]. Therefore, killing cancer cells through ROS or oxidative stress causing-agents represent one of the theories proposed in cancer therapy [21]. In the present study, we showed that both needles and berries extracts lead to a significant increase of H_2_O_2_ content in both cell lines as compared to the negative control (Figure 2A,B). In fact, these extracts have the ability to increase the H_2_O_2_ contents by at least 2-fold compared to the negative control. Lipid peroxidation, which is another indicator of oxidative stress, was quantified by measuring the MDA content (Figure 2C,D). The two methanolic extracts tested in this study, were able to induce an increase in the MDA content compared to the negative control. However, the effect of the needles extract was more pronounced compared to the berries. Moreover, treated MCF-7 cells showed higher levels of MDA compared to the MDA-MB-468 cells.

**Figure 3:**
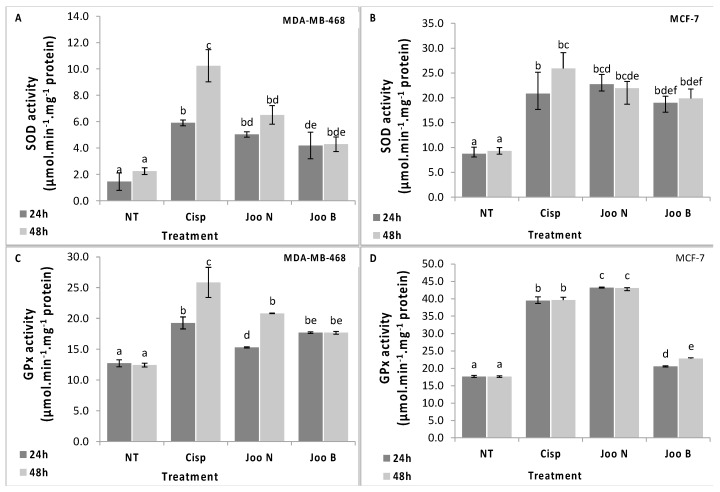
Activities of antioxidative enzymes; superoxide dismutase (SOD, **A** and **B**) and glutathione peroxidase (GPx, **C** and **D**) in the MDA-MB-468 (**A** and **C**) and MCF-7 (**B** and **D**) cell lines after treatment with methanolic extracts of *Joo* needles (*Joo* N) and *Joo* berries (*Joo* B). Each value represents the mean of six replicates. Bars represent the standard error. Different letters indicate significant differences among treatments at *p* = 0.05.

Superoxide dismutase is recognized as the primary defense barrier against ROS which catalyzes the dismutation of superoxide anion radicals (O2^−^) to hydrogen peroxide (H_2_O_2_). Hydrogen peroxide, generated by the activity of SOD is eliminated by its conversion to H_2_O in subsequent reactions by CAT and GPx. The present study demonstrated that the methanolic extracts of *Joo* needles and berries increased significantly the phase I antioxidant enzymes (SOD and GPx) in both cell lines (Figure 3). This increase reached its maximum in the MCF-7 cells treated with the needles extract. The increased levels of antioxidant enzyme activities are considered as a normal strategy from cells to face the oxidative stress caused by the two extracts tested here. However, the impact of the oxidative stress seems to be more pronounced after exposure to the needles and berries extracts. In fact, the determination of lipid peroxidation levels in the cells, measured as MDA content, showed a profound increase in the lipid peroxidation after cell exposure to these extracts for 48 h. This result indicates that despite the increase of antioxidant enzyme activities (SOD and GPx), the oxidative damage was more pronounced that cells failed to stop.

In cancer cells, the intracellular antioxidant capacity is mainly conferred by the glutathione- and thioredoxin-dependent systems. In fact, the important enzymes implicated in the regulation of redox homeostasis such as peroxidases and thiol reductases, are limited to the pool of GSH and thioredoxin as source of reducing equivalents [22]. Thus, to determine the likely cause of the increase in ROS and the reason why cells failed to cope with the oxidative damage after treatment with needles and berries extracts of *Joo*, the effect of the two extracts, on TrxR and GR was evaluated.

**Figure 4:**
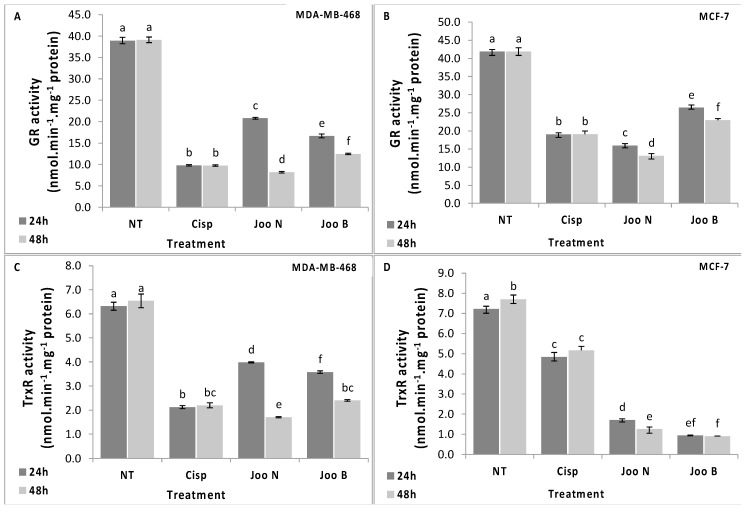
Activities of glutathione reductase (GR, **A** and **B**) and thioredoxin reductase (TrxR, **C** and **D**) in the MDA-MB-468 (**A** and **C**) and MCF-7 (**B** and **D**) cell lines after treatment with methanolic extracts of *Joo* needles (*Joo* N) and *Joo* berries (*Joo* B). Each value represents the mean of six replicates. Bars represent the standard error. Different letters indicate significant differences among treatments at *p* = 0.05.

The results presented here showed that exposure of MDA-MB-468 and MCF-7 tumor cells to methanolic extracts of *Joo* needles and berries differentially affected the GR and TrxR activities (Figure 4). In one hand, both extracts decreased the GR activity compared to the untreated control cells (Figure 4A,B), however, the decrease was more pronounced in the cells treated with needles extracts. In fact, after 48 h exposure, GR activity decreased by 79% and 69% for needles and by 68% and 45% for berries in MDA-MB-468 and MCF-7 cells, respectively. On the other hand, a significant decrease was also noted for the TrxR activity for which the decrease reached 74% and 63% after 48 h treatment of MDA-MB-468 with needles and berries, respectively. However, for MCF-7 cells, the TrxR activity decreased by 82% and 88% after 48 h exposure to needles and berries extracts, respectively. Moreover, the decrease in both enzyme activities was time dependent for both cell lines and both extracts (Figure 4).

It was reported that the inhibition of reduced GSH and thioredoxin synthesis might be used to sensitize cancer cells to chemotherapy agents [23]. Recently, Rodman et al. showed that the depletion of GSH and the inhibition of TrxR activity increased radiation responses in human breast and pancreatic cancer cells [24]. Consequently, the results presented in this study suggest that the methanolic extracts of *Joo* needles and berries have an important anticancer capacity, partially through the increasing of intracellular ROS. These results indicated also that the anticancer activity of both extracts could be due to the inhibition of GR and TrxR activities.

Both glutathione- and thioredoxin-dependent systems require NADPH, as cofactor, to combat ROS generated from oxidative stress [25]. Thus, deficit in the NADPH content may affect the cell response to ROS. In fact, it was reported that cells exposed to oxidative stress exhibited reduced NADPH levels, which may be a result of elevated activity of NADPH-dependent antioxidant systems [26]. NADP+-ICDH is one of the main enzymes implicated in the provision of NADPH [25]. In fact, it was reported that the over-expression of NADP+-ICDH is responsible for high resistance to oxidative stress, however, decreased NADP+-ICDH expression lead to a low resistance [26].

**Figure 5:**
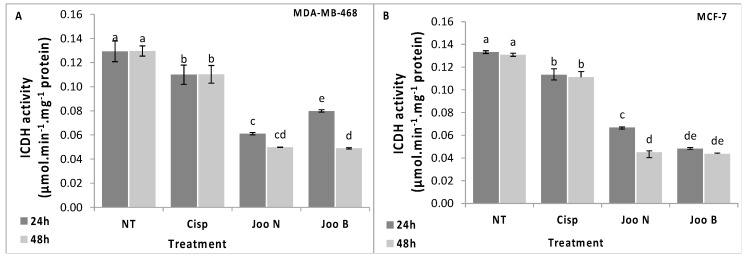
ICDH enzyme activity in the MDA-MB-468 (**A**) and MCF-7 (**B**) cell lines after treatment with methanolic extracts of *Joo* needles (*Joo* N) and *Joo* berries (*Joo* B). Each value represents the mean of six replicates. Bars represent the standard error. Different letters indicate significant differences among treatments at *p* = 0.05.

In this work, exposure of MDA-MB-468 and MCF-7 tumor cells to the methanolic extracts of *Joo* needles and berries led to a significant diminution of the ICDH activity which decreased by 62% for both needles and berries of the MDA-MB-468 treated cells and by 64% and 67% for the needles and berries of the MCF-7 cells after 48 h treatment (Figure 5). The decrease in the ICDH activity may be responsible for the depletion of NADPH, used to cope with oxidative stress through the glutathione- and thioredoxin-dependent systems.

## 3. Materials and Methods

### 3.1. Chemicals and Reagents

All chemicals and biochemicals reagents were purchased from Sigma-Aldrich (Saint Louis, MO, USA) except RPMI 1640 medium and l-glutamine which were from Lonza (Walkersville, MD, USA), fetal bovine serum (FBS) was from Gibco BRL (Cergy Pontoise, France), Bradford reagent was obtained from Bio-Rad (Hercules, CA, USA) and β-nicotinamide adenine dinucleotide 2′-phosphate reduced tetrasodium salt hydrate (NADPH) was purchased from Roche Diagnostics (Mannheim, Germany). The human breast cancer cell lines (MDA-MB-468 and MCF-7) were from the stock cultures of the Laboratory of Experimental Oncology and Natural Substances, Faculty of Sciences & Technology, Sultan Moulay Slimane University (Beni-Mellal, Morocco).

### 3.2. Plant Material and Extracts Preparation

Needles and unripe berries of *Juniperus oxycedrus* subsp. *oxycedrus* were collected in January 2018, from Taza (Morocco). A voucher specimen (No. 1-130118) was prepared, identified by Pr. Abdelali Boulli, (Ph.D, botanist), and deposited at the Laboratory of Biochemistry and Molecular Genetics, Faculty of Sciences and Technologies (Tangier, Morocco).

Air-dried and finely ground plant material (4.5 g of needles or unripe berries without seeds) was added to 45 mL of methanol 80% or dH_2_O and extracted under continuous shaking (250 rpm) and in the dark for 6 h at room temperature. The extracts were filtered with Whatman filter paper and centrifuged at 6000× *g* for 10 min. Then the solvents were evaporated in an incubator at 40 °C. The dried extracts were weighed to determine the percentage yield of the soluble constituents. For needles, the extract yield was 13.26% and 23.31% for aqueous and methanolic extracts respectively. However, in berries, the extract yield was 11.04% for the aqueous extraction and 15.02% for the methanolic extraction.

### 3.3. Total Phenolic Content

The total phenolic content was determined by the method of the Folin–Ciocalteu, following the Singleton and Rossi [27]. To 100 µL of the sample, 400 µL of Folin Ciocalteau’s reagent and 1 mL of saturated Na_2_CO_3 _(7%) were added and the final volume was made up to 1.6 mL with distilled water. The tubes left to stand in the dark for 30 min, after which its absorbance was read at 725 nm against a blank. The total phenolic content of plant extracts was expressed as milligrams of gallic acid equivalents per gram of dry weight (mg GAE/g DW) through the calibration curve with gallic acid.

### 3.4. Total Flavonoid Content

The flavonoid content was assessed following the method of Huang at al. with some modifications [28]. Briefly, 40 µL of each sample was mixed with 10 µL of acetate potassium (1 M) and 10 µL of aluminum chloride (10%). Thereafter, 100 µL of 50% methanol was added and the total volume was made up to 400 µL with distilled water. The absorbance of the mixture was taken at 415 nm. Quercetin was used as standard. The flavonoid content was expressed as milligram of Quercetin equivalence (QE) per gram of extract.

### 3.5. HPLC-DAD Analysis

Reversed phase HPLC method for determination of phenolic acids flavonoids and terpenoids in *Joo* extracts was performed. The analytical HPLC system employed consisted of a Waters 2926 high performance liquid chromatograph system (Waters Corporation, Milford, MA, USA) equipped with a diode array detector. Software used for data acquisition and control of HPLC pumps, auto sampler, and diode array system was Empower 3 (Waters). The separation was carried out on a reversed phase Gemini C6—phenyl column (250 × 4.6 mm, 3 μm) maintained at 30 °C. The mobile phase consisted of two solvents; 0.1% formic acid aqueous solution (A) and methanol (B) operating in gradient form (0 min, 90% A; 10 min, 90% A; 40 min, 65% A; 45 min, 65% A; 60 min, 0% A; 65 min, 0% A; 67 min, 90% A; 75 min, 90% A). The flow rate of the mobile phase was 0.6 mL/min and the injection volumes for all samples and standards were 10 μL. Using different standards, the identification of eluted components was detected spectrophotometrically at 280 nm.

### 3.6. Mineral Content by ICP-AES

ICP-AES was used for rapid and precise determinations of minor and major Mineral content in digested *Joo*. Briefly, 0.5 g of berries or needles powders were digested by heating at 120 °C in 30 mL of concentrated nitric acid HNO_3_. After complete digestion, the mixed sample was cooled at room temperature and made up to a final volume 20 mL with ultrapure water. Digestion was then analyzed in duplicate and concentrations of trace metal elements were measured directly using ICP-AES (Optima 8000 ICP-OES Spectrometer, (PerkinElmer, Inc. Waltham, MA, USA). The optimal instrumental conditions are maintained at 15 L/min for the stable plasma gas flow rate, the auxiliary and the nebulizer gas flow rate was kept at 0.2 and 0.8 L/min, respectively. Indeed, the sample flow rate was 1.5 mL/min and the power was 1500 W.

### 3.7. DPPH Radical Scavenging Assay

The radical scavenging capacity was determined using the stable free radical DPPH (2,2-diphenyl-1-picrylhydrazyl) and following the method described by Hatano et al. with some modifications [29]. Adequate dilutions of sample were realized to obtain a final volume of 50 µL. Extract solutions (50 µL) were mixed with 150 µL of a freshly prepared DPPH solution. The mixture was shaken vigorously and left to stand in the dark and at room temperature for 30 min. The reduction of the DPPH radical was measured at 517 nm. The DPPH scavenging activity was determined by calculating the percentage of DPPH discoloration using the following equation:

%Scavenging effect = [(ADPPH − AS)/ADPPH] × 100(1)

where AS correspond to the values of the sample and ADPPH is referring to the absorbance of the DPPH solution. The extract concentration providing 50% inhibition (IC_50_) was determined from the graph of scavenging effect percentage against extract concentration in the solution.

### 3.8. Radical Scavenging Activity Against the Radical ABTS+

The radical scavenging activity against the radical ABTS+ was evaluated following the method of Re et al. [30]. ABTS+ was generated by the oxidation of ABTS with potassium persulfate. Prior to assay, the ABTS+ stock solution was diluted with methanol until its reach an absorbance of 0.700 ± 0.020 at 734 nm. Then 185 µL of the diluted ABTS+ solution was mixed with 15 µL of the sample and the absorbance was measured at 734 nm after 10 min. The radical scavenging activity was calculated using the following formula:

%Scavenging effect = [(AABTS − AS)/AABTS] × 100(2)

where AS correspond to the values of the sample and AABTS is referring to the absorbance of the ABTS solution. The extract concentration providing 50% inhibition (IC_50_) was calculated from the graph of scavenging effect percentage against extract concentration in the solution.

### 3.9. Metal Chelating Activity

The ferrous ion chelating potential was assessed by the method proposed by Dinis et al. [31]. The reaction mixture is composed of 800 µL of various concentrations of the extracts and 10 µL of FeCl_2_ (0.6 mM). The reaction mixture was shaken vigorously and left to stand at room temperature for 10 min. The reaction was initiated by the addition of 50 µL of ferrozine (5 mM), and the final volume was made up to 1 mL with distilled water. The absorbance of the reaction mixture was measured after 10 min at 562 nm. The control contained all the reagents except sample which is replaced with methanol. The extract concentration providing 50% inhibition (IC_50_) was calculated from the graph of scavenging effect percentage against extract concentration in the solution.

### 3.10. Reducing Power Assay (FRAP)

The reducing power was evaluated following the method of Oyaizu with some modifications [32]. Briefly, 200 µL of sample was mixed with 500 µL of phosphate buffer (0.2 M, pH 6.6) and 500 µL of potassium ferricyanide (1%). Reaction mixture was incubated at 50 ◦C for 20 min and then 500 µL of trichloroacetic acid (10%) was added and centrifuged for 10 min. From the upper layer, 500 µl was mixed with 500 µl of distilled water and 100 µl of ferric chloride (FeCl_3_, 0.1%). Absorbance of all solutions was measured at 700 nm. Values are presented as mg of ascorbic acid equivalent per g of dry weight (mg AAE/g dw).

### 3.11. Cell Culture

MCF-7 (human breast adenocarcinoma) and MDA-MB-468 (triple negative human breast carcinoma) cell line was were maintained with RPMI 1640 medium supplemented with 5% heat-inactivated fetal bovine serum, 1% penicillin G-streptomycin, and 0.2% of l-glutamine. Incubation was performed at 37 °C in humidified atmosphere containing 5% CO2.

### 3.12. MTT Assay

The human breast carcinoma cells MDA-MB-468 and MCF-7 were harvested from starting cultures at the exponential growth phase. After PBS wash, adherent cells were harvested from sub-confluent cultures using cell scraper and suspended in RMPI. The harvested cells were plated at a density of (7 × 10^4^ cells per well) for MDA-MB-468 or (10^5^ cells per well) for MCF-7 in flat-bottomed 96-well microplates containing 100 µL of complete medium and were allowed to adhere overnight before treatment. The cells were treated with several concentrations of *Joo* methanolic extract (needles or berries) from 0.78 to 100 µg/mL, and with cisplatin at concentrations from 0.01 to 25 µg/mL. Control cells were treated with DMSO alone. Extracts dissolved in DMSO completed with medium. The final concentration of DMSO was not exceeded 0.1%. The cells were allowed to grow for 48 h in humidified atmosphere at 37 °C and 5% CO2, then100 µL of medium was carefully removed from each well and replaced with 20 µL MTT solution (5 mg/mL PBS). After 4 h incubation under the same conditions, the cleavage of MTT to formazan by metabolically active cells, which were dissolved in DMSO, was quantified by scanning the plates at 570 nm using a Multiskan EX (Vantaa, Finland) apparatus. Three independent sets of experiments performed in duplicate were evaluated. The % of cell viability was calculated by the following formula:

% Cell Viability = 100 *A/A_0_, (3)

where A_0_ and A are the absorbance of negative control and test culture, respectively. The cytotoxic effects of pyridazin-3(2*H*)-one derivatives against the cell line were compared using their IC_50_ values (concentration of tested molecules leading to 50% inhibition of cell viability).

### 3.13. Cytotoxic Effect against Peripheral Blood Mononuclear Cells (PBMCs)

This test was realized in order to evaluate the effect of methanolic extract of *Joo* needles or berries against non-cancerous cells using the MTT colorimetric assay described below. To isolate the human PBMCs, blood samples were collected from human healthy donors in heparinized tubes and the PBMCs were isolated using standard Ficoll-Hypaque density centrifugation (Biomedical Research Ethics Committee Mohammed University V-Souissi Faculty of Medicine and Pharmacy of Rabat Faculty of Dental Medicine of Rabat 09/06/2014). The interface lymphocytes were washed twice with phosphate buffer solution (PBS). Cells were incubated in 96-well microtiter plates in the presence of the same concentrations of the methanolic extractions parts in the same conditions as tumor cells. The % of Viability calculated using the formula described below.

### 3.14. Clonogenic Formation Assay

Clonogenic assay performed on MCF-7 and MDA-MB-468 tumors cells. The cells were seeded in triplicates on 6 well plates with densities of 500 cells/well plates [33]. After overnight incubation, the cells were treated with 1 µg/mL of *Joo* methanolic extracts neeedles or berries and with 0.1 µg/mL of the respective control cisplatin, then cultured in a 37 °C, 5% CO2 incubator. The media was renewed every 3 days. After 10 days of treatment, the colonies formed were mixed with methanol/acetic acid (7:1), and stained with crystal violet (0.5% *w*/*v*) in methanol for 30 min. The survival fraction was calculated as follows: Surviving fraction = colonies counted/(cells seeded × PE) x100) where PE is the plating efficiency that represents the ratio of the number of colonies to the number of cells plated.

### 3.15. Hydrogen Peroxide (H_2_O_2_) Content Determination

H_2_O_2_ content was determined following the protocol of Hippler et al. [34] with some modifications. Briefly, cells (0.7 × 10^6^ per dish) were seeded in 6-well plates and incubated in complete medium with IC_50_ of both *Juniperus oxycedrus oxycedrus* needles or berries parts extracts. After 48h of incubation, cells were crushed in 0.1% trichloroacetic acid (TCA). The homogenate was centrifuged at 12,000 g for 15 min at 4 °C, the layer was kept in dark for 1 h after mixing with phosphate buffer (10 mM, pH 7.0) and potassium iodide (1 M). The absorbance of the resulting solution was measured at 390 nm. H_2_O_2_ concentrations were calculated using a standard curve.

### 3.16. Malondialdehyde (MDA) Content Determination

Lipid peroxidation measured as malondialdehyde content in MDA-MB-468 cells was determined using thiobarbituric acid (TBA) according to the method described previously [35] with slight modifications. In brief, cell homogenate, in different conditions described above, was mixed with trichloroacetic acid (20%) and TBA (0.67%). The mixture was heated at 95 °C for 1h. After cooling, 1 mL *n*-butanol was added to the mixture followed by centrifugation at 12,000 for 10 min. Organic supernatant was collected to measure the absorbance at 532 nm.

### 3.17. Enzyme Activity Assays

#### 3.17.1. Preparation of Cell Extracts for Antioxidant Enzyme Assays

MDA-MB-468 and MCF-7 tumors cells were treated with *Joo* needles or berries extracts for two times 24 and 48 h, respectively. Then, after washing once with PBS (10mM, pH 7.4), the cells were harvested and centrifuged 1200 g for 10 min. The pellet was suspended in 500 μL of lysis buffer composed of 50 mM Tris-HCl, 1mM phenylmethanesulfonyl (PMSF), 0.1% (*v*/*v*) Triton X-100, in 1.5 mL Eppendorf tubes and maintained in constant agitation at 4 °C for 30 min. The homogenate was then centrifuged (1600 g, 20 min) at 4 °C. The supernatant (enzyme extract solution) was kept at −80 °C or used for the determination of superoxide dismutase (SOD), glutathione peroxidase (GPx), thioredoxin reductase (TrxR), glutathione reductase (GR) and isocitrate dehydrogenase (NADP+-ICDH) activities.

#### 3.17.2. Antioxidant Enzyme Assays

SOD activity was assayed according to the method of Sun et al. [36] with some modifications. Briefly, the reaction mixture was composed of 0.05 M phosphate buffer, pH, 7.5, 10 mM methionine, 0.1 μM EDTA, 2 μM riboflavin, 75 μM nitroblue tetrazolium (NBT) and the enzyme extract. The SOD activity was measured at 560 nm. One unit of SOD activity was defined as the quantity of SOD required to obtain a 50% inhibition of the reduction of NBT. The activity was expressed as units per mg of protein content.

GPx activity was measured by the method of Lawrence and Burk [37] with some modifications. The reaction mixture contained 0.1 M potassium phosphate, pH 7.0, 1 mM EDTA, 1mM sodium azide, 1 mM GSH, GR (10 µg/mL), 0.25 mM NADPH and enzyme extract. The mixture was incubated at 25 °C for 3 min and completed by adding 0.25 mM of H_2_O_2_. The rate of NADPH oxidation was monitored at 340 nm for 5 min. GPx activity was calculated and expressed as nmol of NADPH oxidized/min/mg protein by using the extinction coefficient of 6.2 mM^−1^cm^−1^.

TrxR was measured as the reduction of DTNB (5,5′-dithiobis (2-nitrobenzoic acid)) in the presence of NADPH [38]. The reaction mixture contained 0.1 M phosphate buffer, pH 7.6, 1 mM EDTA, 0.25 mM NADPH, 1 mM DTNB and enzyme extract. The increase in the absorbance at 412 nm was monitored at 25 °C. TrxR activity was expressed as nmol of DTNB reduced/min/mg protein by using the extinction coefficient of 13.6 mM^−1^cm^−1^.

GR activity was estimated by a modified method of Carlberg and Mannervik [39]. Briefly, the reaction mixture contained 0.1 M phosphate buffer, pH 7.6, 1 mM GSSG, 0.2 mM NADPH. The contents were incubated at 25 °C for 3 min and the reaction was initiated by adding enzyme extract. The rate of NADPH oxidation was monitored at 340 nm. GR activity was expressed as nmol of NADPH oxidized/min/mg protein by using the extinction coefficient of 6.2 mM^−1^cm^−1^. NADP+-ICDH activity was estimated according to the procedure of Leterrier et al. [40].

#### 3.17.3. Protein Content Determination

Total protein content of the samples was determined following the method of Bradford [41] using BSA as a protein standard.

### 3.18. Statistical Analysis

The used data are mean values ± S.D. Results were subjected to a one-way analysis of variance (ANOVA) followed by the Tukey test and bivariate correlation was assessed by the test of Pearson using PASW statistics (version 18, Chicago, IL, USA). The differences were considered to be significant when *p* < 0.05.

## 4. Conclusions

In conclusion, we found that the methanolic and aqueous extracts of *Joo* needles and berries are very rich in phenolic metabolites. Interestingly, these extracts exhibited strong antioxidant capacity. Even though, needles have greater amounts of phenolic compounds than the berries, the FRAP assay showed different results and was significantly higher in the berries compared to the needles. This result shows an absence of correlation between this antioxidant test (FRAP) and phenolic and flavonoid contents, and indicates that phenols are not the unique elements responsible for the antioxidant activity.

In order to understand the characteristic of the cytotoxicity effect of the methanolic extract of *Joo* needles and berries on cancer cells, two human breast cancer cell lines were selected to be investigated throughout this study. Analysis of cytotoxic activity, showed potent cytotoxic effects in a dose dependent manner against both breast tumor cells lines (MCF-7 and MDA-MB-468). However, the *Joo* extracts appears to be not cytotoxic towards normal cells (PBMCs).

The investigation of the effect of the methanolic extract of *Joo* needles and berries on the antioxidant system of the two cell lines MDA-MB-468 and MCF-7 showed that ROS generation may be a potential way used to kill both cell lines. We also suggested that the decrease of the enzyme activities of GR and TrxR after treatment with joo extracts may be related the observed cytotoxic effect. According to all the above results, it can be concluded that *Joo* berries and needles extracts could offer a beneficial and natural source of bioactive compounds that can be either used in on the preventive side as used in the clinic without toxicity.

## Figures and Tables

**Table 1:** Phenolic compositions and concentrations of examined *Juniperus oxycedrus* subsp. *oxycedrus* extracts.

Phenolic Compounds mg/100 g dw	*Joo* Needles		*Joo* Berries	
	Aqueous	Methanolic	Aqueous	Methanolic
**Hydroxycinnamic acids**				
**Caffeic acid**	8.68 ± 0.001	5.0 ± 0.001	11.4 ± 0.004	8.1 ± 003
***p*-Coumaric acid**	17.5 ± 0.001	21.2 ± 0.001	ND	ND
**Hydroxybenzoic acids**				
**Gallic acid**	ND	ND	ND	ND
**Salicylic acid**	3398.1 ± 0.026	2942.7 ± 0.017	128.0 ± 0.010	ND
**Syringic acid**	ND	ND	ND	ND
***p*-Hydroxybenzoic acid**	147.7 ± 0.009	416.6 ± 0.006	5.9 ± 0.003w	2.9 ± 0.005
**Flavonoids**				
**Hesperidin**	278.8 ± 0.010	257.2 ± 0.006	147.0 ± 0.013	164.6 ± 0.009
**Naringenin**	64.5 ± 0.004	18.4 ± 0.003	13.8 ± 0.007	19.0 ± 0.001
**Rutin**	1080.5 ± 0.025	160.6 ± 0.007	8.1 ± 0.015	60.0 ± 0.011
**Terpenes**				
**Limonene**	ND	ND	ND	ND
**Thymoquinone**	ND	81.4 ± 0.007	4.3 ± 0.009	37.5 ± 0.006

ND: not detected.

**Table 2:** The levels of mineral contents in needles and berries of *Juniperus oxycedrus* subsp. *oxycedrus*.

Mineral Content mg/kg dw	*Joo* Needles	*Joo* Berries
**Macroelements**		
**Ca**	20,188 ± 1046	4609 ± 164
**K**	7953 ± 188	2778 ± 89
**Mg**	4539 ± 250	3413 ± 109
**Na**	2312 ± 133	2415 ± 219
**P**	1681 ± 170	1613 ± 43
**Microelements**		
**Co**	0.35 ± 0.04	0.37 ± 0.04
**Fe**	285.7 ± 49.07	59.66 ± 2.55
**Mn**	79.91 ± 15.97	29.78 ± 0.57
**Zn**	97.47 ± 16.16	59.01 ± 2.59
**Cr**	0.60 ± 0.17	ND
**Cu**	1.94 ± 0.43	1.91 ± 0.03
**Se**	0.99 ± 0.36	1.28 ± 0.42
**Heavy metals**		
**Cd**	ND	ND

ND: not detected.

**Table 3:** Total phenolic content, flavonoid content and antioxidant activities in aqueous and methanolic extracts of needles and berries of *Juniperus oxycedrus* subsp. *oxycedrus*.

Plant Material	Extraction Solvent	Polyphenols (mg GAE/g dw)	Flavonoids (mg QE/g dw)	Antioxidant Properties (IC_50_ Values; mg/mL)			Reducing Power (mg AAE/g dw)
				DPPH	ABTS	Metal Chelating Activity	
***Joo* N**	Water	147.29 ± 6.76 ^a^	28.66 ± 0.97 ^a^	0.12 ± 0.01 ^a^	0.47 ± 0.04 ^a^	1.53 ± 0.20 ^a^	89.70 ± 4.58 ^a^
	Methanol	292.52 ± 11.68 ^b^	54.58 ± 2.98 ^b^	0.05 ± 0.00 ^b^	0.12 ± 0.00 ^b^	1.59 ± 0.11 ^a^	139.14 ± 2.77 ^b^
***Joo* B**	Water	28.11 ± 3.11 ^c^	3.20 ± 0.79 ^c^	0.96 ± 0.07 ^c^	1.61 ± 0.26 ^c^	0.96 ± 0.03 ^b^	257.99 ± 2.16 ^c^
	Methanol	131.48 ± 4.58 ^d^	8.28 ± 0.74 ^d^	0.09 ± 0.00 ^ab^	0.30 ± 0.01 ^ab^	1.89 ± 0.20 ^c^	941.81 ± 43.21 ^d^

Values are means ± standard deviation of at least six determinations. Different letters in the same column indicate significant differences (*p* < 0.05) within conditions according to Tukey’s multiple range Test.

**Table 4:** IC_50_ values of cytotoxic activity against MDA-MB-468, MCF-7 and PBMCs and % of viability on PBMCs at different concentrations of the extract and CisP. Cells were treated with methanolic extracts of needles and berries from *Juniperus oxycedrus* subsp. *oxycedrus*.

Sample Tested	IC_50_ of Cytotoxic Activity Against Tumor Cells			% of Viability in PBMCs		
	MDA-MB-468	MCF-7	PBMCs	Concentration (µg/mL)		
				12.5	3.125	0.78125
*Joo* N	14.30 ± 3.3 ^a^	10.10 ± 1.40 ^a^	>50 ^a^	91.34 ± 7.72 ^a^	110.40 ± 9.51 ^a^	126.82 ± 10.70 ^a^
*Joo* B	6.40 ± 1.1 ^b^	5.20 ± 2.00 ^b^	49 ^a^	60.40 ± 7.68 ^a^	106.30 ± 1.89 ^a^	118.59 ± 5.56 ^a^
CisP	0.20 ± 0.0 ^c^	2.20 ± 0.40 ^c^	0.27 ^b^	16.08 ± 3.39 ^b^	30.08 ± 3.58 ^b^	37.96 ± 3.44 ^b^

Each value represents the mean ± standard deviation of three independents replicates. Different letters in the same column indicate significant differences (*p* < 0.05) within conditions according to two-way ANOVA multiple comparison range Test.

## Supplementary Materials

The Supplementary Materials are available online. S1: Representative HPLC separations of a mixture of phenolic acids standards at 280 nm. Peak identification: (1) gallic acid (Rt = 6.05); (2) p-hydroxybenzoic acid (Rt = 22.22); (3) caffeic acid (Rt = 29.39); (4) syringic acid (Rt = 33.50); (5) p-coumaric acid (Rt = 39.82); (6) thymoquinone (Rt = 44.90); (7) salicylic acid (Rt = 50.67); (8) rutin (Rt = 51.89); (9) naringenin (Rt = 53.41); (10) hesperidin (Rt = 54.60); (11) limonene (Rt = 59.48). Conditions as described in the text. Conditions as described in the Material and Methods section, Table S1: Correlation coefficients of polyphenols, flavonoids and each antioxidant activity assay, Figure S2: The cytotoxicity effect against the human breast adenocarcinoma MDA-MB-468 and MCF-7 cell lines after treatment at different concentrations with methanolic extracts of needles and berries from *Juniperus oxycedrus* subsp. *oxycedrus* for 48 h. Each value represents the mean ± standard deviation of three independents replicates. Different letters indicate significant differences (*p* < 0.05) within the same concentration, Figure S3: The viability of PBMCs treated at different concentrations of methanolic extracts of needles and berries from *Juniperus oxycedrus* subsp. *oxycedrus* for 48 h. Each value represents the mean ± standard deviation of three independents replicates. Different letters indicate significant differences (*p* < 0.05) within the same concentration.
