# Supplementary material for: Phytochemical Characterization, Antioxidant and In Vitro Cytotoxic Activity Evaluation of Juniperus oxycedrus Subsp. oxycedrus Needles and Berries
Source: Molecules. 2019 Jan 30;24(3):502. doi: 10.3390/molecules24030502 (PMC6384603; doi:10.3390/molecules24030502)
Supplement: Supplementary file 1 [file molecules-24-00502-s001.zip › Supplementary files legends.docx]

**Supplementary Figures Legends**

**Figure S1.** Representative HPLC separations of a mixture of phenolic acids standards at 280 nm. Peak identification: (1) gallic acid (Rt = 6.05); (2) p-hydroxybenzoic acid (Rt = 22.22); (3) caffeic acid (Rt = 29.39); (4) syringic acid (Rt = 33.50); (5) p-coumaric acid (Rt = 39.82); (6) thymoquinone (Rt = 44.90); (7) salicylic acid (Rt = 50.67); (8) rutin (Rt = 51.89); (9) naringenin (Rt = 53.41); (10) hesperidin (Rt = 54.60); (11) limonene (Rt = 59.48). Conditions as described in the text. Conditions as described in the material and methods section.

**Table S1:** Correlation coefficients of polyphenols, flavonoids and each antioxidant activity assay.

**Figure S2.** The cytotoxicity effect against the human breast adenocarcinoma MDA-MB-468 and MCF-7 cell lines after treatment at different concentrations with methanolic extracts of needles and berries from *Juniperus oxycedrus* subsp. *Oxycedrus* for 48 hours. Each value represents the mean ± standard deviation of three independents replicates. Different letters indicate significant differences (p<0.05) within the same concentration.

**Figure S3.** The viability of PBMCs treated at different concentrations of methanolic extracts of needles and berries from *Juniperus oxycedrus* subsp. *Oxycedrus* for 48 hours. Each value represents the mean ± standard deviation of three independents replicates. Different letters indicate significant differences (p<0.05) within the same concentration.
