# Supplementary material for: Phytochemical Characterization, Antioxidant and In Vitro Cytotoxic Activity Evaluation of Juniperus oxycedrus Subsp. oxycedrus Needles and Berries
Source: Molecules. 2019 Jan 30;24(3):502. doi: 10.3390/molecules24030502 (PMC6384603; doi:10.3390/molecules24030502)
Supplement: Supplementary file 1 [file molecules-24-00502-s001.zip › Supplementary Table 1_Ben Mrid.docx]

**Supplementary Table 1.** Correlation coefficients of polyphenols, flavonoids and each antioxidant activity assay.

|  | DPPH | ABTS | metal chelating activity | Reducing power |
| --- | --- | --- | --- | --- |
| Polyphenols | -0.783^*^ | -0.835^**^ | 0.517 | -0.227 |
| Flavonoids | -0.620 | -0.663 | 0.260 | -0.538 |

**. The correlation is significant at the level 0.01 (bilateral).

*. The correlation is significant at the level 0.05 (bilateral).
