# Supplementary figures and images for: Phytochemical Characterization, Antioxidant and In Vitro Cytotoxic Activity Evaluation of Juniperus oxycedrus Subsp. oxycedrus Needles and Berries
Source: Molecules. 2019 Jan 30;24(3):502. doi: 10.3390/molecules24030502 (PMC6384603; doi:10.3390/molecules24030502)

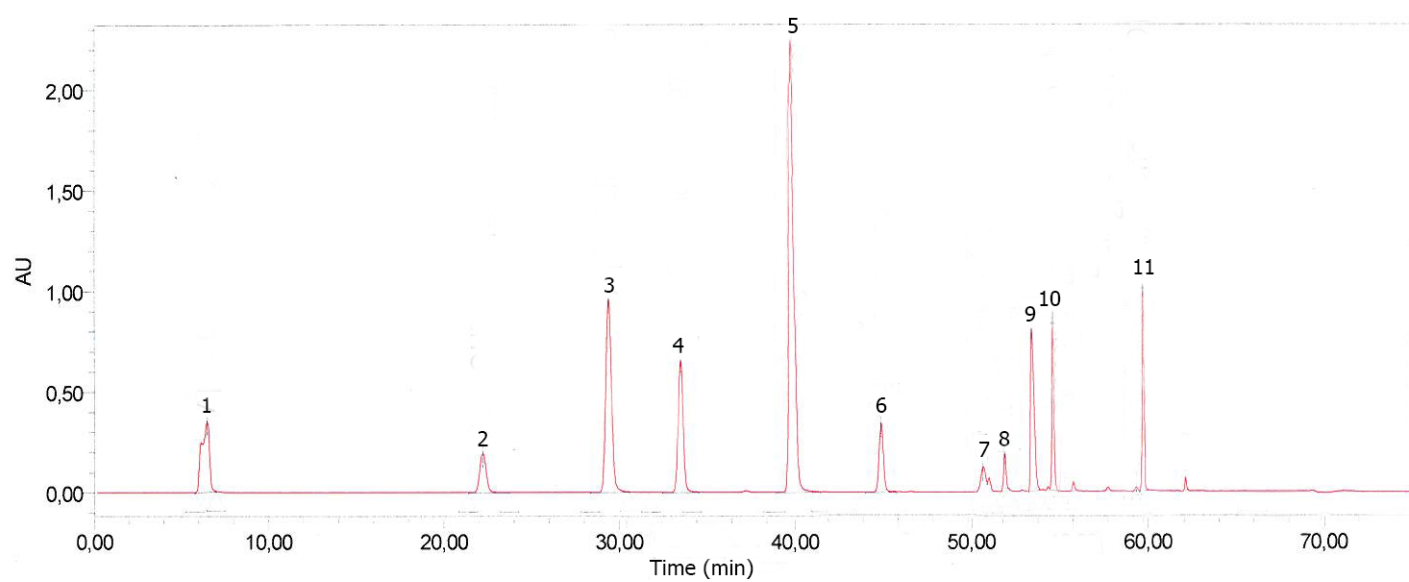

Supplement: Supplementary file 1 [file molecules-24-00502-s001.zip › Supplementary Fig. S1_Ben Mrid.pdf]
